# A Novel Small Molecule FL118 That Selectively Inhibits Survivin, Mcl-1, XIAP and cIAP2 in a p53-Independent Manner, Shows Superior Antitumor Activity

**DOI:** 10.1371/journal.pone.0045571

**Published:** 2012-09-19

**Authors:** Xiang Ling, Shousong Cao, Qiuying Cheng, James T. Keefe, Youcef M. Rustum, Fengzhi Li

**Affiliations:** 1 Departments of Pharmacology and Therapeutics, Roswell Park Cancer Institute, Buffalo, New York, United States of America; 2 Department of Medicine, Roswell Park Cancer Institute, Buffalo, New York, United States of America; 3 NCI-supported Experimental Therapeutics Program, Roswell Park Cancer Institute, Buffalo, New York, United States of America; University of Illinois at Chicago, United States of America

## Abstract

Drug/radiation resistance to treatment and tumor relapse are major obstacles in identifying a cure for cancer. Development of novel agents that address these challenges would therefore be of the upmost importance in the fight against cancer. In this regard, studies show that the antiapoptotic protein survivin is a central molecule involved in both hurdles. Using cancer cell-based survivin-reporter systems (US 7,569,221 B2) via high throughput screening (HTS) of compound libraries, followed by *in vitro* and *in vivo* analyses of HTS-derived hit-lead compounds, we identified a novel anticancer compound (designated FL118). FL118 shows structural similarity to irinotecan. However, while the inhibition of DNA topoisomerase 1 activity by FL118 was no better than the active form of irinotecan, SN-38 at 1 µM, FL118 effectively inhibited cancer cell growth at less than nM levels in a p53 status-independent manner. Moreover, FL118 selectively inhibited survivin promoter activity and gene expression also in a p53 status-independent manner. Although the survivin promoter-reporter system was used for the identification of FL118, our studies revealed that FL118 not only inhibits survivin expression but also selectively and independently inhibits three additional cancer-associated survival genes (Mcl-1, XIAP and cIAP2) in a p53 status-independent manner, while showing no inhibitory effects on control genes. Genetic silencing or overexpression of FL118 targets demonstrated a role for these targets in FL118’s effects. Follow-up *in vivo* studies revealed that FL118 exhibits superior antitumor efficacy in human tumor xenograft models in comparison with irinotecan, topotecan, doxorubicin, 5-FU, gemcitabine, docetaxel, oxaliplatin, cytoxan and cisplatin, and a majority of mice treated with FL118 showed tumor regression with a weekly × 4 schedule. FL118 induced favorable body-weight-loss profiles (temporary and reversible) and was able to eliminate large tumors. Together, the molecular targeting features of FL118 plus its superior antitumor activity warrant its further development toward clinical trials.

## Introduction

Limited efficacy in terms of impact on overall patient survival, toxicity and drug resistance is the major limitation and challenge of present chemotherapy. One way to overcome this challenge is to increase selectivity to cancer and enhance the chemotherapeutic index of anticancer agents. Studies have revealed that the antiapoptotic protein survivin, a unique member in the inhibitor of apoptosis (**IAP**) family, is a pivotal molecule at the junction of cancer cell survival and division networks [1]–[3] and a critical inherent and induced drug/radiation resistance factor for various cancer types during treatment [4]–[12]. The role for survivin in drug/radiation resistance is consistent with its potential role in cancer stem cells (**CSC**) [13]–[16], which are highly resistant to drug treatment [17]. A role for survivin in CSC is independently revealed by computer analysis of the death-from-cancer signature genes. Cancer cells with stem cell-like expression profiles possess three characteristics: increased IAP expression, activated mitotic spindle checkpoint proteins, and elevated expression of cell cycle control proteins [18]. Accordingly, survivin is a key member in the IAP family and possesses all three characteristics: apoptosis inhibition, mitotic control, and cell cycle promotion [4], [19], [20]. Consistent with the CSC concept, while survivin is expressed in all types of cancer, we showed that only a small subset of cancer cells express survivin, and its expression overlapped with several universal stem cell markers including CD133 and ABCG2 [15]. Therefore, development of novel survivin inhibitors may overcome the challenging issues of drug/radiation resistance and cancer relapse.

Although many agents or ligands were reported to inhibit survivin expression, currently, there are only two survivin inhibitors in development. One is YM155, a survivin expression suppressant found to specifically inhibit survivin expression and show antitumor activity in preclinical animal models [21], [22]. Inhibition of survivin expression by YM155 is at least partially via its inhibition of survivin transcription [23]. YM155 is currently in Phases I/II clinical trials [24], [25]. The other inhibitor, shepherdin, is a survivin_79–87_ peptidomimetic agent that interrupts HSP90-survivin interactions, and thus destabilizes survivin [26]–[28]; this is the first example of proof of principle. Further development of shepherdin toward clinical trials has not been reported, which could be due to known inherent issues for peptidomimetics in drug production and delivery. Additionally, two promising survivin antisense oligonucleotides were identified [29], [30]. Both ISIS 23722 (LY2181308) and SPC3042 are currently in clinical trials. Together, it appears that developing small molecule chemical inhibitors targeting survivin with high antitumor efficacy and low toxicity is highly desirable for cancer treatment.

Survivin is a multifunctional molecule with unique multi-subcellular localizations in cancer cells. Survivin has been shown to associate with both mitotic spindles [31] and centromeres [32], [33] during mitosis, [34] as well as in mitochondria [35]. Its expression is involved in inhibition of apoptosis, [31], [35] and regulation of mitotic cell division, [29], [32], [33], [36] as well as in promotion of the G_1_/S transition, [37], [38], in which the survivin-controlled and p53-dependent expression of p21 may play a role [39], and regulation of gene transcription [39], [40]. In order to overcome potential challenges for one agent to inhibit multiple functions of survivin, we generated cancer cell-based high throughput screening (HTS) assays in which the regulatory sequence of the survivin gene driving a luciferase reporter gene was stably expressed in cancer cells as a drug selection marker for finding compounds that alter the expression of survivin [41]. Using the genetically engineered compound-screening assay, we performed HTS with several compound libraries including compounds in the public domain collected by the National Cancer Institute (NCI). Here we report the *in vitro* and *in vivo* characterization of a lead compound (designated FL118) identified via HTS of compound libraries, followed by analysis of HTS-derived initial hit-relevant analogs. Our data indicate that FL118 selectively targets several cancer-associated survival genes (survivin, Mcl-1, XIAP and cIAP2) and shows superior antitumor activity in a p53 status-independent manner. These features warrant its further development toward clinical trials.

## Materials and Methods

### Ethics Statement

All procedures relevant to the use of animals and animal models of human tumor xenograft in this study were approved by the Institutional Animal Care and Use Committee (IACUC) of Roswell Park Cancer Institute, and are consistent with the National Institutes of Health Guide for Care and Use of Laboratory Animals.

### Cell Lines and Cell Culture

The human head and neck squamous cell carcinoma cell line FaDu (p53 mutant), the human ileocecal adenocarcinoma cell line HCT-8 (p53 wild type), the colon cancer cell line SW620 (p53 mutant), the human colorectal carcinoma cell line HCT116 (p53 wild type), the human prostate cancer cell lines PC-3 (p53 null) and LNCaP (p53 wild type), the human breast adenocarcinoma cell line MCF7 (p53 wild type), the human lung carcinoma cell line A549 (p53 wild type), and human non-cancerous cell lines HGF (human gingival fibroblast, p53 wild type) and AHDF (adult human dermal fibroblast, p53 wild type) were from American Type Culture Collection (ATCC, Manassas, VA). The human ovarian cancer cell line 2008 (p53 mutant, a gift from Dr. Kunle Odunsi, Roswell Park Cancer Institute) was derived from a patient with ovary cystadenocarcinoma [42]. The human lung cancer cell line EKVX (p53 mutant, a gift from Dr. Daniel Chan, University of Colorado Health Sciences Center) [43] was originally from National Cancer Institute [44]. Cells were grown either in RPMI 1640 Medium (HCT-8, SW620, HCT116, PC-3, LNCaP, MCF7, A549, EKVX, and 2008, HGF and AHDF) or in Eagle's Minimum Essential Medium (FaDu), which was supplemented with 10% heat inactivated FCS, 100 U/ml of penicillin and 0.1 µg/ml of streptomycin. Cells were cultured in a 5% CO2 incubator at 37°C and subcultured every 3–5 days. All cell lines were mycoplasma-free confirmed using MycoSensor PCR Assay kit (Stratagene).

### Drug Screening Models and Compound Libraries for High Throughput Screening

Cancer cell-based high throughput screening (HTS) assay model systems that used the survivin gene regulatory sequence driving a luciferase reporter gene were generated in our laboratory [41]. Briefly, a 4080 bp human survivin promoter starting from the 3′ end of the ‘A’ base in the translation start site (ATG) of the human survivin gene was cloned upstream of the luciferase reporter gene. After Sal I linearalizing the vector backbone, the entire survivin promoter-luciferase reporter cassette was stably expressed via G418 selection in several types of cancer cell lines derived from colon (HCT116), lung (A549), breast (MCF7), prostate (PC-3), and ovary (2008). The genetically engineered cancer cell clones were then individually tested for luciferase modulation using model ligands, hedamycin [6] and Hoechst 33342 [5], to validate individual cancer cell clones. The validated cell clones were expanded and used for compound library screening. Specifically, model cells were seeded in 96 well plates (2000 cells per well) overnight. Different compounds in DMSO were then added into individual wells containing the model cells to a final concentration of 1 µM for 24 hours, and DMSO only and model ligands were used as negative and positive controls. After treatment, cells were tested for luciferase activity. Of note, the 1 µM for 24 hours treatment condition for individual drugs is important for the initial screening to get the true hits via our drug screening assay system without resulting in a significant number of false positive hits. This is because 1) if a compound could significantly exert its inhibitory effect on survivin promoter activity, the 1 µM for 24 hours treatment condition would be sufficient to observe an effect, while this treatment condition may not significantly induce cancer cell death; and 2) many compounds in the library could significantly induce cancer cell death if a higher concentration and/or longer time treatment was used (e.g. 10 µM for 24 hours or 1 µM for 48 hours) and thus resulting in false positive hits. In short, the use of a minimal and sufficient drug concentration and treatment time that match the nature of the drug screening assay system is the key to find true hits in the initial screening step and thus, this saves time for further evaluation of the identified hits. Drug screening was performed in triplicate to monitor consistency and to avoid false positives. The initially identified hits from one cell model (HCT116-luc) were further cross-tested in the other four cell models described above (A549-luc, MCF7-luc, PC-3-luc, 2008-luc). The hits that produced consistent effects in different cancer cell types were further studied *in vitro* and *in vivo*.

The HTS assay systems were used for the screening of candidate compounds that alter the expression of survivin. The libraries of small chemical compounds used during drug screening were from multiple sources including compounds in the public domain collected by the National Cancer Institute (NCI) Developmental Therapeutics Program (DTP). The compound libraries collected by NCI include the “Structural Diversity Set”, which contains 1,990 structural representative compounds and was derived from and represented ∼140,000 non-redundant compounds available on the plates, the “GI Diversity Set” which contains 879 representative compounds and was derived from and represented 37,863 non-redundant compounds that have been tested in the NCI human tumor 60-cell line screen, and the “Mechanistic Challenge Set”, which contains less than a hundred compounds.

### Compound Screening Processes via HTS

**Figure 1:**
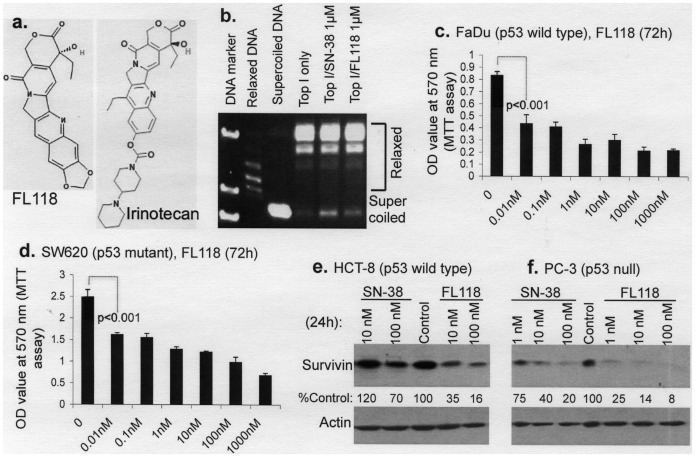
Distinct effects of FL118 on DNA topoisomerase 1 (Top1) activity and cancer cell growth. **a.** Structure of the camptothecin analogs FL118 (MW: 392) and of irinotecan (MW: 587). **b.** FL118 inhibits DNA Top1 to a similar extent as the active form of irinotecan SN-38. A representative result tested at 1 µM (the highest concentration that can be reached by irinotecan *in vivo*) is shown. Experiments were performed as described in Methods. **c** and **d.** FL118 effectively inhibits cancer cell growth at sub-nM levels. Cancer cells at 50–70% confluence were treated with vehicle (DMSO) or FL118 at different concentrations as shown in **c** and **d**. Cell growth/viability were determined by MTT assay 72 hours after treatment. Each bar is the mean ± SD (standard deviation) derived from independent assays (N = 4) performed in at least triplicate. Representative statistical p-values are indicated. **e** and **f.** Comparison of the effects of FL118 and the irinotecan active form SN-38 on survivin expression in two cancer cell types with different p53 status. Subconfluent cancer cells with distinct p53 status were treated with or with SN-38 or FL118 for 24 hours as shown. Cells were then lysed and analyzed using Western blots with corresponding antibodies as shown (e, f). Actin was used as internal controls.

Diverse small chemical compounds were initially screened using the genetically engineered cancer cell models described above. The initial screening resulted in about 250 hit candidates which showed inhibition of luciferase activity in drug-treated model cells at a concentration of 1 µM for 24 hours. Several consecutive rounds of screening of the 250 hit candidates using a series of different concentrations (from 0.001 nM to 1000 nM) resulted in 20 top-hit compounds, which showed inhibition of luciferase activity in a concentration range of 1 nM to 100 nM within 24 hours of treatment. We further analyzed 207 chemical structure analogs relevant to the 20 hit compounds for their ability to inhibit survivin promoter activity in parallel with determination of cancer cell growth inhibition by each compound *in vitro* via MTT assay. We found that FL118 shows strong inhibition of survivin promoter activity. The chemical definition of FL118 is “10H-1,3-Dioxolo[4,5-g]pyrano[3',4':6,7]indolizino[1,2-b] quinoline-8,11(7H,12H)-dione, 7-ethyl-7-hydroxy-,(S)-”. Its CID number is 72403 and its NSC number is 634724. Other synonyms related to FL118 chemical definition include, but may not be limited to, “7-ethyl-7-hydroxy-(7S)-7,8,11,13-tetrahydro-10H- [1], [3]dioxolo[4,5-g]pyrano[3',4':6,7]indolizino[1,2-b]quinoline-8,11-dione”; “(7S)-7-ethyl-7-hydroxy-10H- [1], [3]dioxolo[4,5-g]pyrano[3',4':6,7]indolizino[1,2-b]quinoline-8,11(7H,13H)-dione”; “10H-1,3-Dioxolo(4,5-g)pyrano(3',4':6,7)indolizino(1,2-b)quinoline-8,11(7H,13H)-dione, 7-ethyl-7-hydroxy-, (±)-”; and “10H-1,3-Dioxolo(4,5-g)pyrano(3',4':6,7)indolizino(1,2-b)quinoline-8,11(7H,13H)-dione, 7-ethyl-7-hydroxy-, (7S)-”. Its structure is shown in Figure 1a.

### DNA Topoisomerase 1 Activity Assay

Inhibition of DNA topoisomerase 1 (Top1) activity by FL118 was determined using a Top1 Assay Kit from TopoGEN (Port Orange, Florida) following the protocol in the kit by checking FL118’s ability in blocking Top1 activity to convert supercoiled plasmid DNAs into relaxed plasmid DNAs. Each reaction contains one unit of Top1, 0.625 µg supercoiled plasmid DNA substrates, 10 mM Tris-HCl (pH7.9), 1 mM EDTA, 150 mM NaCl, 0.1% bovine serum albumin (BSA), 100 µM spermidine and 5% glycerol. The reaction was supplied with or without FL118 or SN-38 (the active form of irinotecan). Incubations were carried out at 37°C for 30 minutes and the reaction was stopped by adding 5 µl stop buffer/gel loading dyes (provided in the kit). The plasmid DNAs in each reaction were then separated on a 1% agarose gel, then stained with ethidium bromide and visualized under an ultraviolet light.

### MTT Cell Growth and Viability Assay

Cancer cell growth/viability was determined by MTT assay [45]. MTT, a tetrazolium salt with the chemical definition of 3-[4,5-dimethylthiazol-2-yl]-2,5-diphenyltetrazolium bromide, was used as a colorimetric substrate for measuring cell viability. Seventy-two hours post treatment with vehicle or with FL118, MTT was added to a final concentration of 0.5 mg/ml into treated cells in 96-well plates. Cells were further incubated in a 5% CO_2_ incubator at 37°C for 4 hours, and then lysed thoroughly with lysis buffer (20% SDS, 50% N, N-dimethylformamide, pH 4.7, 100 µl/well). The absorbance in the relevant wells was measured at 570 nm using an Ultra Microplate Reader (Bio-Tek Instruments). Alternatively, cell survival after FL118 treatment was monitored by trypan blue exclusion. Surviving cells were counted using the Vi-Cell™ XR Cell Viability Analyzer (Beckman Coulter) 72 hours after treatment, and cell viability was calculated as a percentage.

### Antibodies and Expression Vectors Used

Survivin (FL-142) and p21^WAF1/CIP1^ (C-19) antibodies were purchased from Santa Cruz. Actin antibody (A2066) was purchased from Sigma. DHFR polyclonal antibody was home made and a gift from Dr. Bruce Dolnick (Roswell Park Cancer Institute, Buffalo, NY). Antibodies for Mcl-1, Bcl-2, Bcl-XL, XIAP, cIAP2, Bax, Bim, active/cleaved caspase-3 and PARP were purchased from Cell Signaling. pcDNA3-XIAP-Myc (plasmid 11833), pcDNA3.1-hcIAP2 (plasmid 8338) [46], pCMV-Flag-hMcl-1 (plasmid 25392), and pGL2-Mcl-1 (plasmid 19132) [47] were purchased from Addgene (www.addgene.org). The pcDNA3-XIAP was a gift from Dr. Dario C. Altieri (Wistar Institute Cancer Center). pcDNA3-survivin was characterized previously [31].

### Western Blot Analysis and Quantification

Western blot analysis of protein expression was performed following our previous protocols [48]. Briefly, cells with or without FL118 treatment were washed with PBS (50 mM phosphate pH 7.4, 100 mM NaCl, 10 mM KCl) and lysed on ice for 30 minutes in PBS containing 1% Nonidet P-40, 0.5% sodium deoxycholate, 0.1% SDS, 10 µg/ml PMSF, and 20 µM leupeptin. After the lysates were cleared by centrifugation at 15,000 g for 20 minutes at 4°C, the total protein was determined using Bio-Rad protein assay solution. Up to 50 µg of total protein was denatured in 2 X SDS sample loading buffer for 5 minutes at 95°C, separated on 10–15% SDS-PAGE gels, and electrotransferred to the Pure Nitrocellulose Membrane (Bio-Rad, Hercules, CA) using semi-dry electrophoretic transfer. After the nonspecific binding sites on the membranes were blocked with 5% skim milk in TBS-T (20 mM Tris-HCl pH 7.5, 0.137 M NaCl, and 0.01% Tween 20) for 3 h at room temperature with constant shaking, the membranes were incubated in TBS-T containing the relevant primary antibody (1∶500–1000) and 5% BSA overnight at 4°C. After washing with TBS-T three times, the membrane was incubated in 5% skim milk in TBS-T buffer containing the appropriate secondary anti-IgG antibody (1∶5000) at room temperature for 1 h with constant shaking. The protein of interest was detected using Western Lightning®-ECL (Perkin Elmer, Waltham, MA) and visualized by exposure for various times (5–60 seconds). For normalization of protein loading, the same membranes were stripped with stripping buffer (100 mM 2-mercaptoethanol, 2% sodium dodecyl sulphate, 62.5 mM Tris-HCl pH 6.7) and used for Western blot by the same procedure with actin antibody (1∶1000 dilution). The actin result was used as an internal control. The protein bands of interest on the Western blot images were quantitated using the Personal Densitometer SI and ImageQuant5.2 Software (Molecular Dynamics). After the quantitative data from each protein of interest were normalized to the corresponding actin data, the normalized data were then calculated into a percentage change relative to the no treatment control, which was set at 100 for antiapoptotic proteins or set at 1 for proapoptotic proteins. The umbers less then one were not counted.

### BrdU Labeling Proliferation Assay and Flow Cytometry

Bromodeoxyuridine (5-bromo-2'-deoxyuridine, BrdU) is an analogue of thymidine and is commonly used to detect cell proliferation. To evaluate the inhibitory effect of FL118 treatment on cancer cell growth, the BD Pharmingen™ BrdU Flow Kit was used following the manufacturer’s instructions. HCT-8 cells were treated with and without (control) FL118 for 24h at 10 nM and labeled with BrdU (10 µM) during the last 40 minutes of FL118 treatment. The cells were then collected and incubated in BD cytoperm permeabilization buffer plus for 10 min on ice, followed by fixation/permeabilization with BD cytofix/cytoperm buffer for 30 min at room temperature. To expose incorporated BrdU, the cells were treated with DNase (300 µg/ml) for 1 hour at 37°C following re-fixation in BD cytofix/cytoperm buffer for 5 min. Subsequently, cells were resuspended in 50 µl of BD Pwrm/Wash Buffer containing diluted fluoresecent anti-BrdU antibody (1∶100) and rinsed with 1 ml of 1×BD Perm/Wash Buffer. Finally, the cells were stained with 7-AAD solution before flow cytometry analysis. BrdU-positive labeled cells were analyzed using WinList 3D (version7.1) and the histogram was plotted using Excel 2010.

### Luciferase Activity Assay

Cells were seeded in 48-well plates (∼2.5×10^4^ per well) and grown to sub-confluence in complete cell culture medium in all experiments. Cells were either stably transfected with the pLuc-4080 survivin promoter-luciferase construct or transiently transfected with relevant luciferase reporter vectors. For transient transfection, 245 ng of targeting luciferase reporter construct plus 5 ng of internal control vector, pRK-tk in 30 µl serum-free DMEM, was mixed in a 1.5 ml tube containing 30 µl serum-free DMEM containing 0.4 µl Lipofectamine™ 2000. After incubation at room temperature for 20–25 minutes, the DNA/Lipofectamine complex was added to each well of 48-well plates, which already contained 300 µl corresponding complete growth medium. The DNA/Lipofectamine complex was replaced after incubation for 16 hours by complete growth medium containing either DMSO or FL118. Cells were further incubated for an additional 24 hours, followed by processing luciferase assays. For luciferase assay, a Dual-Luciferase Reporter Assay System (Promega) was used. Cells in 48-well plates were washed with PBS and lysed with 80 µl 1×passive lysis buffer on a shaker for up to 1 hour at 4°C. Twenty µl cell lysate per well was used to measure the Firefly and Renilla luciferase activity in triplicates in a Luminometer by subsequently adding 20 µl luciferase assay reagent and 20 µl Stop-Glo reagent. Data were normalized to Renilla luciferase activity (internal control) as arbitrary units to show relative promoter activity.

### Quantitative Real-time PCR (Real-time qPCR)

Total RNA was extracted from cells using TRI REAGENT RT (Molecular Research Center, Inc, Cincinnati, OH). Total RNA (2 µg per sample) was converted to cDNA using anchored oligo (dT) primers (RevertAid First Strand cDNA Synthesis Kit, Thermo Scientific) following the manufacturer’s instructions. Individual reverse transcription reactions in a total volume of 20 µl were then diluted into 200 µl with ddH2O. Ten µl was used for real-time qPCR using the iTaq SYBR Green Supermix with ROX (Bio-Rad, Hercules, CA). The sequences of oligonucleotides (primers) used in real-time qPCR reactions for Bax and Bim were: 5′-AGGATGCGTCCACCAAGAAG-3′(Bax-F2, forward) and 5′-CCAGTTGAAGTTGCCGTCAGA -3′ (Bax-R2, reverse) for amplifying Bax product [49]; 5′-GCCCVACCTGCCAGC-3′(Bim-F1, forward) and 5′-ACAGCAGGGAGGATCTTCTCATAA-3′ (Bim-R1, reverse) for amplifying Bim product [50]. GAPDH was used as an internal control. The primer pair for GAPDH was provided in the RevertAid First Strand cDNA Synthesis Kit. Triplicate qPCR reactions were performed for each of the samples, and 3 samples were tested in each condition of no treatment, treated with 10 nM FL118 and treated with 100 nM FL118 in parallel. The real-time qPCR condition is 95°C for 3 min as a pre-denature step, followed by 40 PCR cycles at 95°C for 15 sec and 60°C for 45 sec. The data were analyzed using the Applied Biosystems 7300 Real Time PCR System and normalized to GAPDH.

### HDAC Activity Assay

The measurement of HDAC activity was performed using an EpiQuiktm HDAC activity/inhibition assay kit (ET Epigentek, Farmingdale, NY) according to the manufacturer’s instructions. Ten million SW620 colon cancer cells at 90% confluence were harvested and nuclear extracts were prepared as described previously [6], [23]. For determination of HDAC activity, 10 µg nuclear extracts with or without FL1118 at different concentrations or HDAC inhibitor (HDACi, positive control) were added to each strip well which contains stably captured biotinylated acetylated histone substrate. Samples were incubated at 37°C for 60 minutes to let HDACs bind to and deacetylate histone substrate. Subsequently, the high affinity acetylated histone antibody (1 µg/ml) was used to recognized un-deacetylated substrate. The amount of the un-deacetylated substrate is inversely proportional to HDAC enzyme activity. Finally, the enzymatic activity of HDACs was detected using a microplate reader at 450 nm following an ELISA-like reaction. HDAC activity was expressed as relative OD values per hour per mg of protein sample (OD/h/mg).

### Preparation of Lentiviral Survivin shRNA and Mcl-1 shRNA Infection Particles

The preparation of lentiviral infection particles followed the protocol described in our recent publication [39]. In this study, survivin shRNA (V2LHS_262484) [51] and Mcl-1 shRNAs (V2LHS_72721 location 172_0554-H-8) in the pGIPZ lentiviral vectors (with Puromycin for selection and TurboGFP as visual marking of shRNA expressing cells) in the bacterial stock from the GIPZ lentiviral shRNAmir library (RPCI shRNA Facility in collaboration with Open Biosystems) were prepared using midi prep kits. The HEK 293T packaging cells at 80% confluence were incubated for 24 hours at 37°C and 5% C02 and transfected by gently replacing the cell medium with 500 µl DNA/Lipo complex with gently swirling. Three ml of 293T media (DMEM with 10% FBS and 1% Pen/Strep) were added after a few minutes at room temperature for 16 hours at 37°C and 5% CO2. The 500 µl DNA/Lipo complex was prepared as follows: 250 µl DMEM containing 2.5 µg pGIPZ shRNA, 2.5 µg psPAX2 (or pCMV-dR8.74), 1.0 µg pMD2.G in one tube were mixed with 250 µl DMEM containing 9–12 µl lipofectamine and kept at room temperature for 20 minutes. The medium in the transfected HEK 297T cells in the dish was replaced with new 293T media the next day, and the dish was incubated for an additional 24 hours at 37°C with 5% CO2. Virus-containing supernatant was harvested and filtered through a 0.45 µm cellulose acetate (low protein binding) syringe filter, and the virus stored at 4°C. The TurboGFP expression was checked before collection of the virus in the supernatant. The transfected 293T cells in the dish were incubated with another 3 ml 293T media at 37°C with 5% CO2 overnight. The supernatant collected as above was then combined with the second batch supernatant together as viral stock, which were stored at 4°C for the experiments.

### Infection of Target Cells with Lentiviral Particles

Infection of target cells with lentiviral stock was carried out following a previous protocol [39]. Briefly, HCT-8 colon cancer cells grown to sub-confluence in 6-well plates were infected with 1 ml lentiviral stock prepared as above in the presence of 4 µg/ml polybrene. In order to increase cell infection rates, the plate was spun at 1800 rpm for 45 minutes at room temperature on a microtiter rotor. The infected cells in the plate were further incubated for 3–6 hours and then an additional 1 ml cell media was added and cells were cultured overnight. Cells in individual wells were then diluted five times (one plate to five plates) and incubated for 24 hours, followed by selection with puromycin (2 µg/ml) for 3–7 days. The resultant puromycin-selected cells were directly used for the experiments.

### Transfection of XIAP and cIAP2 Expression Vectors into HCT-8 Colon Cancer Cells

HCT8 cells were transfected with expression vectors for XIAP, cIAP2 or empty vector using Lipofectamine™ 2000 (Invitrogen) following the manufacturer’s instructions. Briefly, cells were transfected with 1.5 µg plasmid DNA mixed with 2.5 µl Lipofectamine™ 2000 for one 6-well plate containing sub-confluent cells (4×10^5^ cells/well). Twenty-four hours after transfection, the transfected cells were treated with FL118 (10 nM) for 36 h. Cells were then used for either western blot analysis or Annexin V/PI staining followed by flow cytometry analysis. Transfection experiments were performed at least in triplicate for each experiment.

### Annexin V/propidium Iodide (PI) Double Staining and Flow Cytometry

Apoptosis induced by FL118 treatment was alternatively identified by flow cytometry using Alexa Fluor 488 (or 647) Annexin V/Dead Cell Apoptosis Kit (Invitrogen, Grand Island, NY). XIAP or cIAP2 expression vectors were transfected into HCT8 cells. The transfected cells were treated with FL118 for 36 h at 10 nM. Cells were then collected, washed with PBS, and resuspended in 1× Annexin-binding buffer up to 1×10^6^ cells/ml. Subsequently, 5 µl Alexa Fluor 488 Annexin V and 1 µl 100 µg/ml PI were added to 100 µl of the cell suspension. After 15 min incubation in the dark at room temperature, 400 µl of 1× binding buffer were added to each tube. The resultant samples were immediately analyzed by flow cytometry. The experiment examining apoptosis after silencing of survivin with or without FL118 treatment was the same as above with the exception that Alexa Fluor 647 was used instead of 488 to avoid interferences from EGFP (infection marker control). In this situation, HCT-8 cells were infected with lentiviral survivin-shRNA particles instead of transfection of expression vectors. In both cases, fluorescence parameters were gated using unstained control cells and 10,000 cells were counted for each sample. The data were analyzed using WinList 3D (version7.1) and the histogram was plotted using Excel 2010.

### FL118 Stock and Working Solution Preparation for *in vitro* and *in vivo* Experiments

For *in vitro* studies, FL118 was dissolved in DMSO at 1 mM as a stock solution. Immediately prior to addition of FL118 to the cells, the stock solution was further diluted with DMSO to a concentration of 1000× the final concentration used for the experiment. The 1000× working stock solution was directly diluted into experiment-relevant buffers or cancer cell type-relevant media. For *in vivo* studies, FL118 was first dissolved in DMSO at a concentration of 1 mg/ml (2.55 mM) and further diluted in freshly made saline containing Tween-80 to make a final working solution containing 0.05 mg/ml (0.128 mM) FL118 (W/V), 75% saline (V/V), 20% Tween-80 (V/V) and 5% DMSO (V/V). Control solution (placebo or vehicle) was 75% saline, 20% Tween-80 and 5% DMSO without FL118. All other drugs (irinotecan, topotecan, doxorubicin, 5-FU, gemcitabine, docetaxel, oxaliplatin, cytoxan and cisplatin) were commercially formulated ready for clinical application and purchased from the Pharmacy section of the Hospital at Roswell Park Cancer Institute. All drugs were administered by intraperitoneal injection routes (i.p.).

### Determination of the Maximum Tolerated dose of FL118 with the Weekly × 4 Schedule

The maximum tolerated dose (MTD) of FL118 was determined using athymic nude female mice at 6–12 weeks of age by starting with 0.5 mg per kg per week (mg/kg/wk) for four weeks; we escalated doses by 0.25 mg/kg each time until MTD was achieved. Each dose was tested on a cohort of 5 mice in individual independent experiments. For some doses, we made one or two additional repeats. MTD was defined as the highest dose that could be given resulting in no drug-related moribund state or death, while temporary body weight loss was within 20%. Other signs of toxicity considered in the experiment included mouse behavior, movement and diarrhea. During MTD testing, mice were monitored for signs of toxicity defined above. Six to 12-week-old female athymic nude mice (nu/nu, body weight 20–25 gm) were obtained from Charles River Laboratories International, Inc. (Wilmington, MA) or Harlan Sprague Dawley Inc. (Indianapolis, IN).

### Animal Models of Human Tumor Xenografts

Two types of human cancer xenografts were used in the studies: 1) from human cancer cell line-established xenografts [using nude or severe combined immunodeficiency (SCID) mice]; 2) from a cancer patient tumor tissue-established xenograft (primary tumor-derived xenograft, using SCID mice), which was previously established and characterized [52]. Human tumor cell line-derived xenografts were initially established by subcutaneously injecting 1×10^6^ cultured cancer cells. The derived tumors were then passed several generations in nude mice by transplanting 40–50 mg non-necrotic tumor mass via a trocar after the xenograft tumor reached ∼1000 mm^3^. Cancer cell line-derived tumors used in this study included human FaDu (squamous cell carcinoma) head-&-neck tumor, human HCT-8 (ileocecal adenocarcinoma) colon tumor, and human SW620 colon tumor. Treatment was initiated 7 days after tumor transplantation when the tumor reached 200–250 mm^3^, at which time the treatment was designated as Day 0. All cancer cells used for tumor establishment were mycoplasma-free. The transplantable tumor has a similar histological profile to that of the patient’s tumor. Six to 12-week-old female SCID mice were obtained from the Roswell Park Animal facility. Mice were housed 5 mice per cage with water and food *ad libitum*. All animal experiments were performed in accordance with IACUC-approved animal protocols.

### Tumor Measurement and Antitumor Activity Analysis

Two axes (mm) of a tumor (L, longest axis; W, shortest axis) were measured with a Vernier caliper. Tumor volume (mm^3^) was estimated using a formula of “tumor volume = ½ (L×W^2^)”. Tumor measurement was taken daily for the first four weeks with the exception of weekends and then three times weekly for the following two weeks of post treatment and twice weekly thereafter. Antitumor activity of an agent was assessed by maximum tumor growth inhibition (MTGI), which is the mean tumor volume difference between the treated group (MTWTG) and the untreated control group (MTWCG) at the same time point. The calculated formula is “MTGI = (MTWTG–MTWCG)÷MTWCG×100%”. The tumor doubling time (TDT) was defined as the mean time for the tumor to reach twice its initial volume from the time (defined as Day 0) at which mice began treatment. Tumor response was analyzed using the following parameters. 1) Partial tumor response (PR), which was defined as when tumor volume was reduced to at least 50% of the initial tumor size on Day 0; and 2) complete tumor response (CR) which was defined as the inability to detect tumor at the initial site of tumor transplant. Cure was defined as mice achieving complete tumor regression for ≥30 days after the termination of drug treatment.

### Statistical Analysis

Statistical significance among the mean values was analyzed via an unpaired two-tailed Student t-test assuming equal variance. The significance (p-value) was set at the nominal level of 0.05 or less. Each bar or time point is presented as mean ± standard deviation (SD) from the *in vitro* data. The figure data from the *in vivo* animal studies are presented as mean ± standard error (SE). The data in the Tables from the animal studies are presented as mean ± SD.

## Results

### Inhibition of Cancer Cell Growth by FL118 is Significantly Greater than its Ability to Inhibit DNA Topoisomerase 1 Activity

**Figure 2 pone-0045571-g002:**
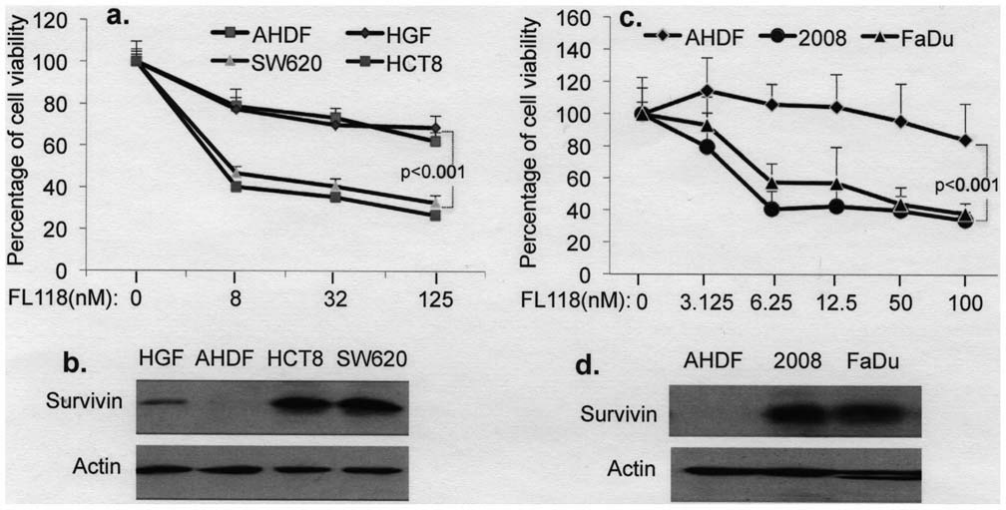
Cancer cells with high survivin expression are significantly more sensitive to FL118 treatment than non-cancerous cells, which have low or no survivin regardless of p53 status. Cells at about 50% confluence were treated with or without FL118 in a series of concentrations as shown (**a**, **c**) for 72 hours. Cell growth/viability were then determined using MTT assay and plotted as percentage cell viability curves. Alternatively, cells at 80–90% confluence were lysed and analyzed using Western blots for survivin expression (**b**, **d**). **a.** Cell viability curve for two cancer cell lines (SW620, HCT-8) versus two non-cancerous cell lines (AHDF, HGF) after FL118 treatment for 72 hours at the different concentrations as shown. **b.** Comparison of the expression of survivin among two cancer cell lines (SW620, HCT-8) versus two non-cancerous cell lines (AHDF, HGF). Actin was used as an internal control. **c.** Cell viability curve for additional two cancer cell lines (2008, FaDu) versus one non-cancerous cell line (AHDF) as a control after FL118 treatment for 72 hours at different concentrations as shown. **d.** Comparison of the expression of survivin in additional two cancer cell lines (2008, FaDu) versus one non-cancerous cell lines (AHDF) as a control. Actin was used as an internal control. The data on each data point shown in **a** and **c** are the mean ± SD derived from at least three independent assays in triplicate.

**Figure 3:**
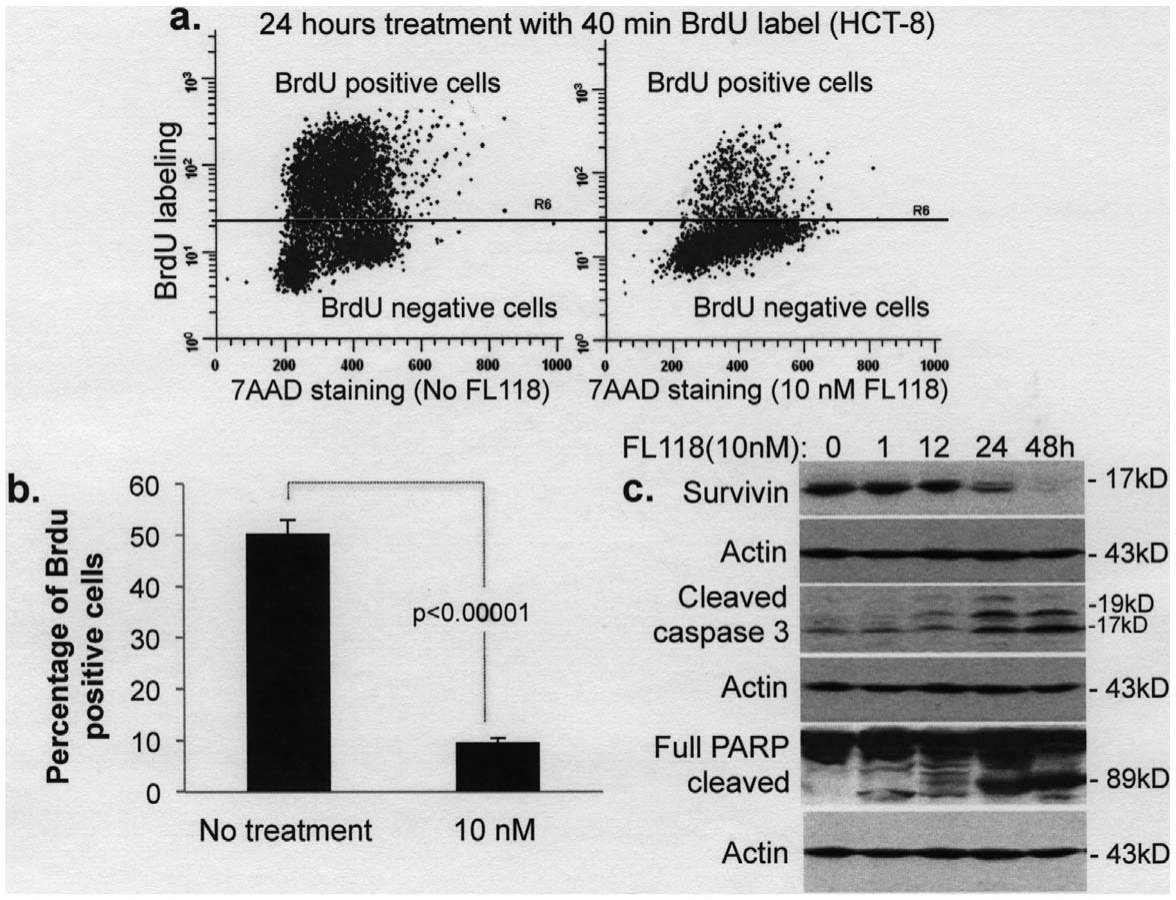
Treatment of cancer cells with FL118 significantly decreases BrdU positive cells (proliferative cells) and induces apoptotic markers. HCT-8 cells at 50% confluence were treated with or without FL118 (10 nM) for 24 hours and were labeled with BrdU in the last 40 min of FL118 treatment. Cells were then stained with 7AAD, followed by flow cytometry analysis. **a.** A representative example is shown. **b.** Statistical results derived from three independent flow cytometry analyses. **c.** FL118 induces caspase-3 activation and PARP cleavage, hallmarks of apoptosis. Sub-confluent HCT-8 colon cancer cells were treated with or without FL118 at the concentration and time points shown. Cells were then lysed and analyzed by western blot. Actin expression was used as an internal control. Quantification as %Control: 100, 101, 93, 21, 4 for survivin; 1, 1, 2, 8, 13 for activated caspase 3; and 1, 2, 3, 31, 49 for cleaved PARP.

**Figure 4:**
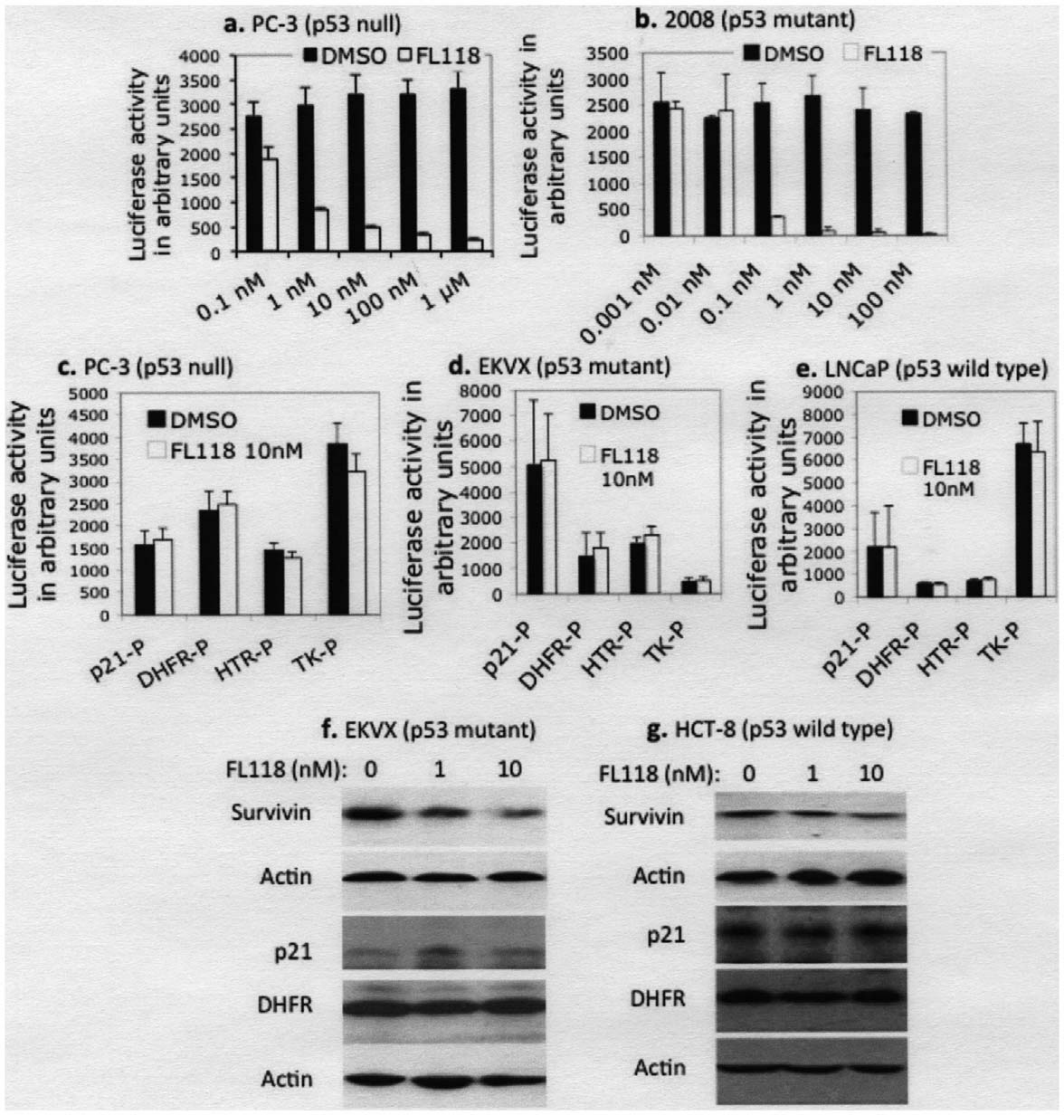
FL118 selectively inhibits human survivin promoter activity and endogenous survivin expression. **a** and **b**. FL118 inhibits survivin promoter activity at concentrations as low as 0.1–1 nM. Cancer cells that stably express a full-length survivin promoter (4080 bp)-driven luciferase construct were treated with FL118 for 24 hours at different concentrations as shown, followed by luciferase activity assay. **c**, **d** and **e**. FL118 does not inhibit promoter activity for the genes of p21, DHFR, HTR or TK at 10 nM levels. Sixteen hours after transfection, cells were treated with FL118 (10 nM) for 24 hours, followed by luciferase activity assay. Each bar (**a–e**) is the mean ± SD derived from independent testing (N  = 3) of at least triplicates. **f** and **g**. FL118 inhibits endogenous survivin expression but does not inhibit expression of endogenous p21 and DHFR proteins. Representative results are shown. Subconfluent EKVX (**f**) and HCT-8 (**g**) cells were treated with and without FL118 for 24 hours, then cell lysates were analyzed by Western blot. Actin expression was used as an internal control. Quantification: **f.** %Control: 100, 32, 15 for survivin; 100, 255, 165 for p21; and 100, 91, 97 for DHFR. **g.** %Control: 100, 48, 32 for survivin; 100, 95, 99 for p21; and 100, 101, 112 for DHFR.

As shown in Figure 1a, FL118 and irinotecan are structurally similar and belong to the camptothecin (CPT) analog family. It is known that the major mechanism of action (MOA) for irinotecan is to inhibit DNA topoisomerase 1 (Top1) activity [53]–[57]. We therefore compared the effect of FL118 with SN-38 (the *in vitro* active form of irinotecan) on the inhibition of DNA Top1 activity at different concentrations. The results indicated that FL118 is not a better DNA Top1 inhibitor than SN-38. Here we show the results derived from the highest concentration of SN-38 (1 µM) that can be reached by irinotecan *in vivo* (Fig. 1b). In contrast, FL118 inhibits cancer cell viability (Fig. 1c, d and Fig. 2a, c), proliferation (Fig. 3a, b) and survivin promoter activity (Fig. 4) as well as induces apoptosis (Fig. 3c) at nM or sub-nM levels. We therefore propose that the inhibition of DNA Top1 activity by FL118 does not significantly contribute to its ability to inhibit cancer cell growth and proliferation. In order to determine the potential difference between FL118 and the irinotecan active form SN-38, we further compared their effects on survivin expression. Our data suggest that FL118 is a better inhibitor for survivin than SN-38 overall (Fig. 1e, f).

### Cancer Cells with High Survivin Expression are Significantly More Sensitive to FL118 Treatment than Non-cancerous Cells which have Low or Undetectable Survivin Expression

To determine a differential association of survivin expression with sensitivity to FL118 treatment between cancer cells and non-cancerous cells, we first chose two colon cancer cell lines with different p53 status (HCT-8 with wild type p53 and SW620 with mutant p53) and two non-cancerous cell lines (AHDF and HGF, both with wild type p53) for comparison. We found that the two cancer cell lines with high survivin expression were much more sensitive to FL118-mediated cell growth inhibition than the two non-cancerous cells regardless of p53 status (Fig. 2a, b). To further confirm this differential association, we tested two additional cancer cell lines (FaDu with wild type p53 and 2008 with mutant p53) using the AHDF non-cancerous cell line as a control. Similar results were obtained (Fig. 2c, d). These results suggest that the efficacy of FL118 is independent of p53 status, but do not definitively address how the expression levels of survivin modulate the effectiveness of FL118, since the results only showed an association/correlation. However, this issue is address more rigorously below using genetic approaches.

### FL118 Treatment of Cancer Cells Inhibits Proliferation and Induces Apoptosis

We next alternatively determined the effect of FL118 on cancer cell growth and apoptosis. Treatment of cancer cells with FL118 for 24 hours significantly inhibited cell proliferation determined by BrdU label and flow cytometry experiments (Fig. 3a, b). Consistent with this and other (Figs. 1, 2) observations, cancer cells treated with FL118 induced caspase 3 activation and PARP cleavage, hallmarks of apoptosis (Fig. 3c),

### FL118 Inhibits Survivin Promoter Activity and Endogenous Gene Expression but Shows No Inhibitory Effects on Control Genes

Next, we investigated the selectivity of FL118 in the inhibition of survivin promoter activity. Consistent with its initial discovery using the survivin promoter-driven luciferase reporter system as a biomarker/target, and the data shown in the Figures 1–3, FL118 selectively inhibited human survivin promoter-driven luciferase activity at concentrations as low as 0.1–1 nM in various cancer cell types regardless of p53 status (Fig. 4a, b). In contrast to its inhibitory effects on survivin promoter activity, FL118, even at a 10 nM level, showed no inhibitory effects on luciferase activity driven by control promoters of the cyclin-dependent kinase inhibitor p21^WAF1/CIP1^ (p21), dihydrofolate reductase (DHFR), human thrombin receptor (HTR), or thymidine kinase (TK) genes in various cancer cell types with different p53 status (Fig. 4c–e). Consistently, determination of endogenous protein expression in EKVX and HCT-8 cancer cell lines showed that p21 and DHFR protein levels were not inhibited by FL118, while survivin was inhibited by FL118 (Fig. 4f, g). These observations indicate the selectivity of FL118 in inhibiting the expression of the survivin gene.

### FL118 also Modulates Expression of Other Protein Members of the IAP and Bcl-2 Families

**Figure 5:**
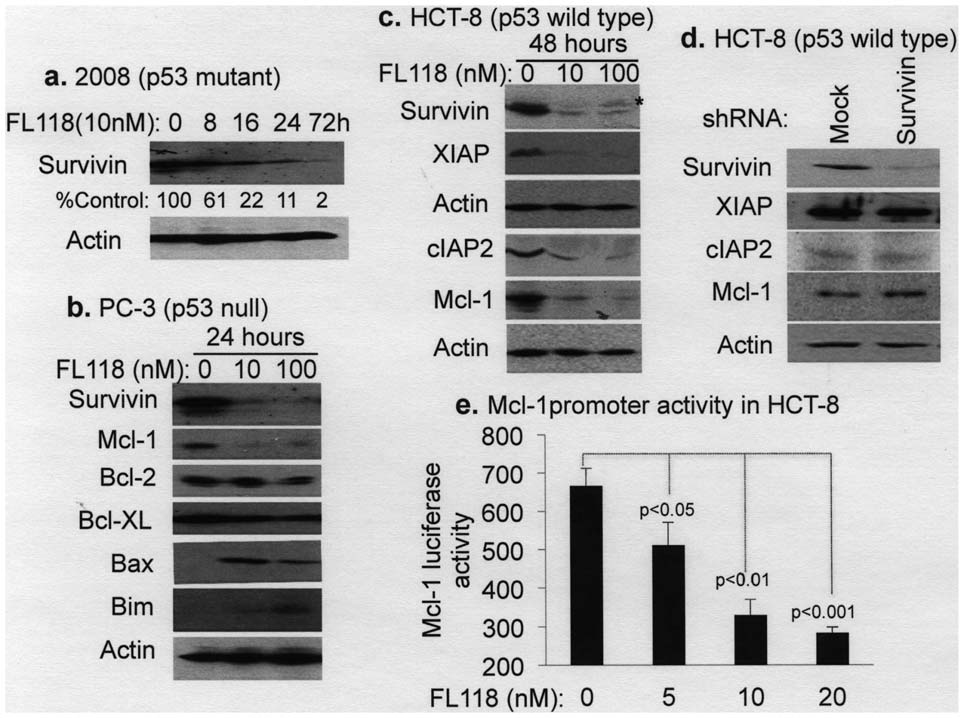
FL118 selectively and differentially modulates the expression of IAP and Bcl-2 family proteins. Subconfluent cells were treated with and without FL118 at the concentration and time points as shown (**a**–**c**). Cells were then lysed and analyzed by western blot. Representative results are shown. **a.** A time course for FL118-mediated inhibition of survivin expression in 2008 ovarian cancer cells. **b.** Differential modulation of the expression of antiapoptotic and proapoptotic proteins by FL118 in PC-3 prostate cancer cells. Quantification as %Control: 100, 7, 5 for survivin; 100, 9, 12 for Mcl-1; 100, 88, 77 for Bcl-2; 100, 54, 49 for Bcl-XL; 1, 100, 43 for Bax; 1, 18, 74 for Bim. **c.** Downregulation of survivin, XIAP, cIAP2 and Mcl-1 by FL118 in HCT-8 colon cancer cells. The “*” in the survivin panel indicates the potential expression of the proapoptotic protein survivin-2B after FL118 treatment. Quantification as %Control: 100, 11, 7 for survivin (of note, the asterisk-marked band excluded); 100, 9, 8 for XIAP; 100, 22, 12 for cIAP2; and 100, 13, 6 for Mcl-1. **d.** Genetic silencing of survivin does not affect the expression of other FL118 downstream targets. HCT-8 colon cancer cells were infected with lentiviral survivin-shRNA for 48 hours, followed by western blot analysis of the expression of FL118 downstream targets. Quantification as %Control: 100, 13 for survivin; 100, 96 for XIAP; 100, 98 for cIAP2; and 100, 136 for Mcl-1. Of note, the survivin level without mock lentiviral infection is similar to the mock control lentiviral infected cells (not shown). Actin expression shown in **a–d** is internal controls. **e.** FL118 inhibits Mcl-1 promoter-driven luciferase activity. Subconfluent HCT-8 colon cancer cells were transiently transfected with pGL2-Mcl-1. The transfected cells were treated with or without FL118 for 24 hours 16 hours post transfection. Each bar is the mean ± SD derived from three independent assays.

**Figure 6:**
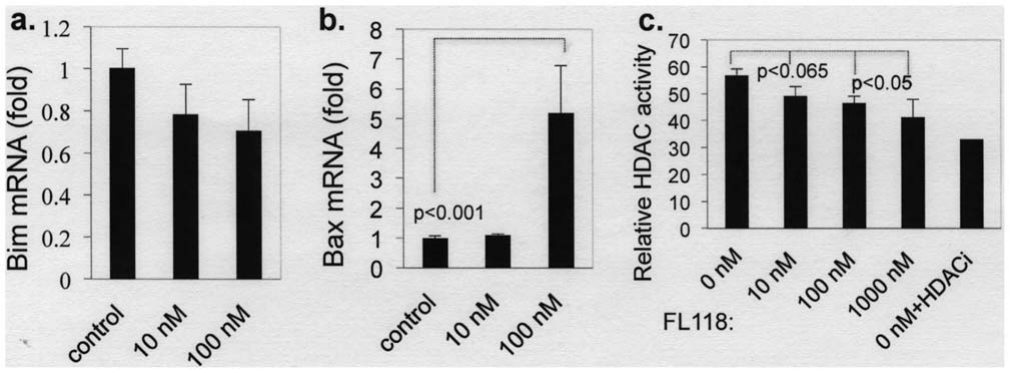
Effect of FL118 on Bim and Bax mRNA and HDAC activity. **a** and **b.** Effect of FL118 on Bim and Bax mRNA. Subconfluent HCT-8 cells were treated with or without FL118 for 24 hours, followed by isolation of total RNA for real-time qPCR using either Bim primers (a) or Bax primers (b) following the procedures described in the Methods. Data is plotted as a histogram. Each bar is the mean ± SD derived from three independent assays in triplicate. **c.** Effect of FL118 on HDAC activity. Nuclear extracts were isolated from subconfluent SW620 cells. The HDAC activity inhibition experiment was performed following the protocol in the Method section. The resultant data were plotted in a histogram; each bar is the mean ± SD derived from three independent assays.

Further studies revealed that FL118 also selectively modulates the expression of several other protein members in the IAP and Bcl-2 families. Specifically, our data revealed that treatment of cancer cells with FL118 results in the downregulation of Mcl-1, XIAP and cIAP2 in addition to survivin in a p53 status-independent manner, while FL118 treatment showed minimal effects on Bcl-2 and Bcl-XL (Fig. 5a–c). In contrast, FL118 treatment increased the expression of pro-apoptotic proteins (Bax, Bim, Fig. 5b), possibly including the pro-apoptotic survivin-2B, marked by an asterisk (top panel in Fig. 5c*), indicating the differential and selective effects of FL118 on the expression of cancer survival-and-death-associated proteins. We further demonstrated that genetic silencing of survivin using lentiviral survivin-shRNA systems results in no inhibitory effects on the expression of other FL118 downstream targets (Mcl-1, XIAP or cIAP2, Fig 5d), suggesting that FL118-mediated inhibition of Mcl-1, XIAP and cIAP2 is independent of its inhibition of survivin. Consistent with this observation, we showed that FL118 also inhibits Mcl-1 promoter-driven luciferase activity (Fig. 5e). These findings suggest that one potential strategy for FL118 to control its downstream target gene expression is to inhibit their transcription. To further explore the mechanism of FL118-mediated induction of Bim and Bax proteins (Fig. 5b), we performed real-time qPCR. Our data showed that FL118 does not increase Bim mRNA (Fig. 6a), while significantly increasing Bax mRNA at 100 nM (Fig. 6b). Interestingly, our data further demonstrated that FL118 inhibits HDAC activity at its 100 and 1000 nM concentration (Fig. 6c). These data suggest that induction of Bim and Bax protein expression by FL118 likely uses both transcriptional and post-transcriptional mechanism.

### FL118 Downstream Targets (Survivin, Mcl-1, XIAP, cIAP2) Play a Role in FL118-induced Cancer Cell Growth and Apoptosis Inhibition

**Figure 7:**
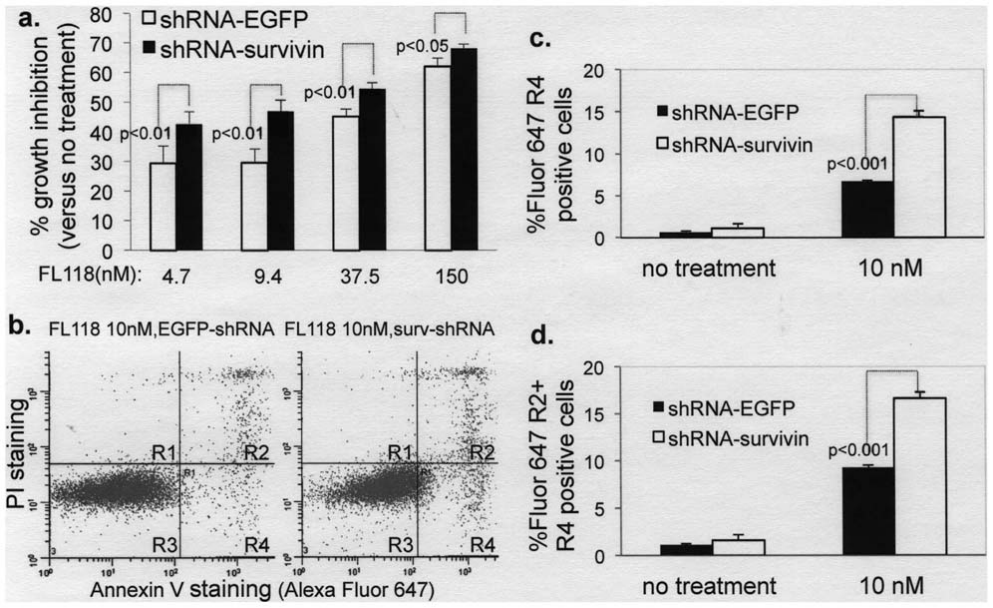
The FL118 downstream target survivin play a role in FL118-mediated inhibition of cancer cell growth and apoptosis. **a** Subconfluent HCT-8 colon cancer cells were infected with a lentiviral delivery system containing mock shRNA (shRNA-EGFP) or survivin shRNA. After puromycin selection at 2 µg/ml, the puromycin-selected infectants were treated with or without FL118 for 72 hours. Cells were then analyzed using the MTT assay for cell viability. Each bar is the mean ± SD derived from three independent assays. Of note, downregulation of survivin by the lentiviral survivin shRNA was confirmed by western blots (Fig. 5d). **b–d.** Subconfluent HCT-8 colon cancer cells were infected with lentiviral survivin shRNA particles or control lentiviral particles as above. After up to 7 days selection with puromycin (2 µg/ml), the mixed infectants were treated with or without FL118 (10 nM) for 36 hours. Cells were stained with Annexin V/PI, followed by flow cytometry. **b.** A representative flow cytometry result gated with PI (Y axis) and Annexin V (Alexa Fluor 647, X axis). **c** and **d.** Quantitative data from **b** for R4 (c) and R2+R4 (d) from three independent measures in parallel. Of note, R1 is Annexin V negative/PI positive cells; R2 is both Annexin V and PI positive cells (later apoptotic cells); R3 is both Annexin V and PI negative cells; and R4 is Annexin V positive/PI negative cells (early apoptotic cells).

We have shown that cancer cells with high survivin expression are more sensitive to FL118-mediated growth inhibition in comparison with non-cancerous cells that show lower survivin expression (Fig. 2). Since these data were derived from different cell types, a definitive role for survivin expression in FL118 sensitivity could not be concluded. To evaluate this more directly, we used survivin shRNA to knock down survivin expression in HCT-8 cells (Fig. 5d shows survivin knockdown). When these cells were treated with FL118, growth inhibition by FL118 versus the no treatment control was significantly higher in survivin knockdown cells in comparison with the EGFP control (Fig 7a), suggesting that in these cells, survivin plays a role in reducing FL118 effects. Alternatively, we further performed Annexin V staining and flow cytometry experiments after lentiviral survivin shRNA to knock down survivin in cancer cells in the presence and absence of FL118 treatment. Our data from these experiments demonstrated that silencing of survivin increased Annexin V staining in comparison with FL118 treatment alone. A representative result was shown in Figure 7b, and the statistical analysis of these data was shown in Figure 7c, d.

**Figure 8:**
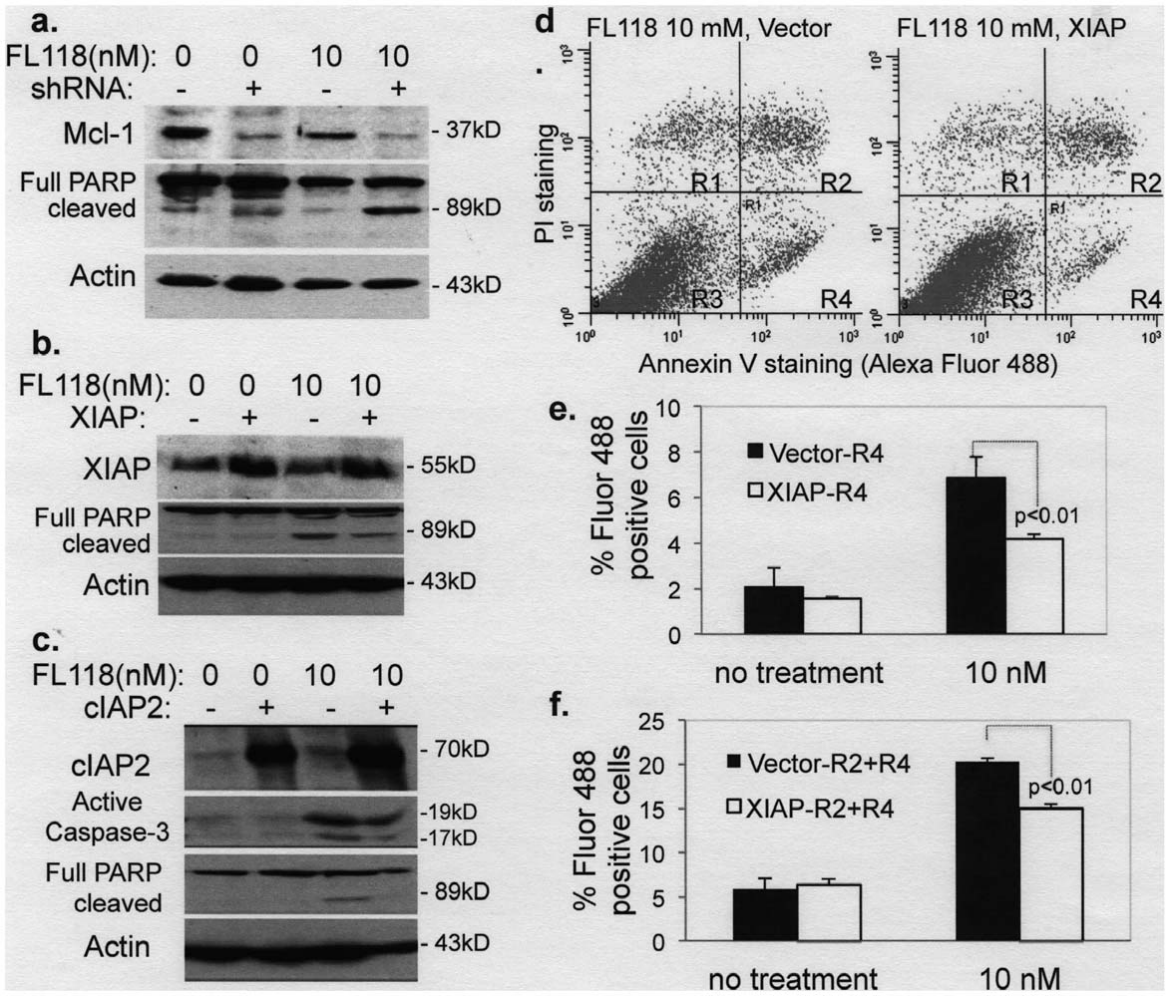
FL118 downstream targets (Mcl-1, XIAP, cIAP2) play a role in FL118-mediated apoptotic marker production and apoptosis. **a** Subconfluent HCT-8 colon cancer cells were infected with a lentiviral delivery system containing mock shRNA (shRNA-EGFP) or Mcl-1 shRNA. After puromycin selection, the puromycin-selected infectants were treated with or without FL118 for 36 hours. Cells were then analyzed using western blot for PARP cleavage. Quantification as %Control: 100, 8, 57, 6 for Mcl-1; 1, 2, 1, 18 for cleaved PARP. **b** and **c.** Subconfluent HCT-8 colon cancer cells were transfected with or without mammalian expression vector for XIAP (b) cIAP2 (c) overnight, and then treated with or without FL118 (10 nM) for 36 hours. Cells were then lysed and analyzed via western blots to determine caspase-3 activation or PARP cleavage. Quantification as %Control: **b.** 100, 247, 100, 244 for XIAP; 1, 2, 16, 7 for cleaved PARP. **c.** 100, 1470, 100, 1645 for cIAP2; 1, 1, 21, 15 for activated caspase 3; and 1, 1, 3, 1 for cleaved PARP. Actin expression was used as internal controls (**a–c**). **d–f.** Effect of overexpression of XIAP on FL118-induced Annexin V staining (apoptosis). Subconfluent HCT-8 colon cancer cells were transfected with XIAP expression vectors for 16 hours. The transfected cells were then treated with or without FL118 (10 nM) for 36 hours. Cells were stained with Annexin V/PI, followed by flow cytometry. **d.** A representative flow cytometry result gated with PI (Y axis) and Annexin V (Alexa Fluor 488, X axis). **e** and **f.** Quantitative data from **d** for R4 (e) and R2+R4 (f) from three independent measures in parallel. Of note, same as in Figure 7, R1 is Annexin V negative/PI positive cells; R2 is both Annexin V and PI positive cells (later apoptotic cells); R3 is both Annexin V and PI negative cells; and R4 is Annexin V positive/PI negative cells (early apoptotic cells).

We further examined the potential role of other proteins, namely Mcl-1, XIAP and cIAP2 in the sensitivity of cancer cells to FL118. As can be seen in Figure 8a–c, knockdown of Mcl-1 increased, while overexpression of XIAP or cIAP2 inhibited FL118-mediated apoptotic signaling. Interestingly, we failed to observe a significant caspase-3 activation decrease after XIAP overexpression (not shown). Similar to our approach used above, we alternatively performed Annexin V/PI staining and flow cytometry experiments to further determine the role of XIAP in FL118-induced apoptosis. Our data demonstrated that overexpression of XIAP in cancer cells significantly decreased Annexin V staining (Fig. 8d–f), suggesting the protective role of XIAP in FL118-induced apoptosis. Together, these data support the notion that FL118 downstream targets play distinct mechanistic roles in FL118-induced cancer cell killing.

### The Maximum Tolerated Dose (MTD) for FL118 is about 1.5 mg/kg in the Schedule of Weekly × 4

After determining the efficacy of FL118 in cell culture, the next step was to determine whether FL118 had antitumor activity *in vivo*. The first step was to determine the MTD for FL118 and to do so, we followed the protocol described in the Methods. Since FL118 has structural similarity to irinotecan (Fig. 1a), and since the clinically relevant schedule for irinotecan is weekly × 4, we selected the weekly × 4 schedule to determine the MTD for FL118. Table 1 summarizes the MTD data for FL118 in nude mice with the weekly × 4 schedule. As shown, the MTD for FL118 at the defined schedule is about 1.5 mg/kg (Table 1).

**Table 1:** The maximum tolerated dose (MTD) of FL118 at the schedule of weekly × 4.

Drug	Dose(mg/kg)	Schedule	Mouse No^1^	Weight loss	Lethality (%)
Control	0	i.p. weekly × 4	20	6.0±2.5	0
FL118	0.50	i.p. weekly × 4	5	9.8±5.7	0
FL118	0.75	i.p. weekly × 4	10	9.1±3.8	0
FL118	1.00	i.p. weekly × 4	15	12.2±4.8	0
FL118	1.25	i.p. weekly × 4	10	12.1±6.8	0
FL118	1.50	i.p. weekly × 4	15	15.1±3.7	0
FL118	1.75	i.p. weekly × 4	5	29.0±6.7	80

1 Five athymic nude mice per group were used for independent individual experiments. Data are means ± SD.

### FL118 Shows Superior Antitumor Activity in Mouse Models of Human Colon and Head-&-neck Cancer Xenograft Tumors

**Figure 9:**
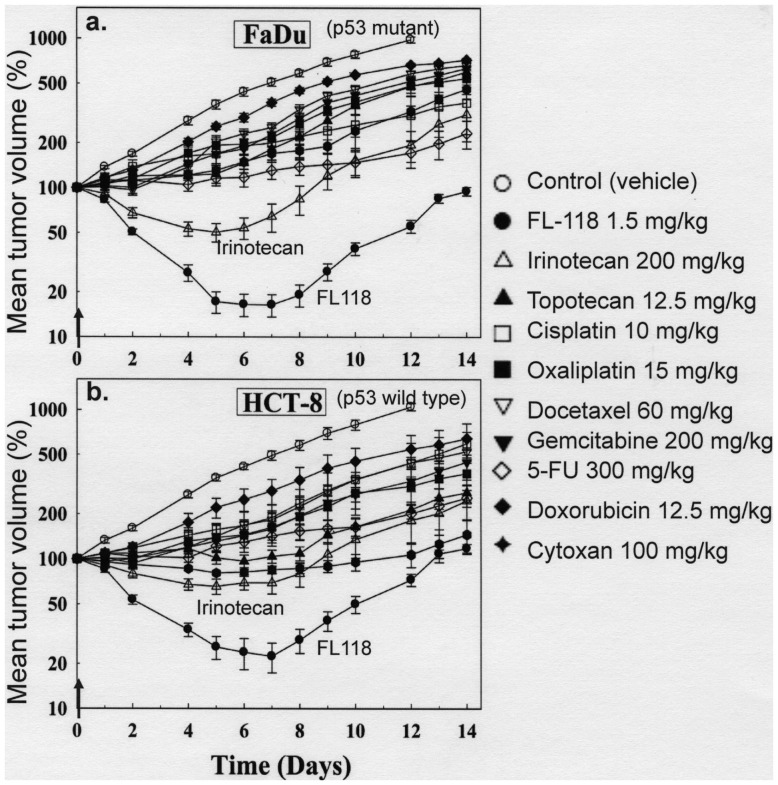
A single injection of FL118 shows superior antitumor activity in comparison with other clinically used antitumor drugs. **a**. Results from the human head and neck FaDu tumor xenograft mouse model. **b**. Results from the human colon tumor HCT-8 xenograft mouse model. Treatment was initiated 7 days after subcutaneous tumor implantation, at which time the tumor volume was about 200–250 mm^3^ (designated Day 0). Drugs were intraperitoneally (i.p.) administrated on Day 0 (indicated with arrow) at their MTD with a single dose schedule. The dose for FL118 is its MTD for the weekly × 4 schedule (Table 1). The tumor growth curves in response to each compound are shown as the mean tumor size ± SE from five individual mice at each time point.

Next, we studied the *in vivo* antitumor activity of FL118 using animal models of human FaDu head-&-neck and HCT-8 colon tumor xenografts, which are the most appropriate models for irinotecan. We compared the antitumor activity of FL118 with clinically used chemotherapeutic drugs including irinotecan and topotecan (DNA Top1 poison), cisplatin and oxaliplatin (DNA platinating agents), docetaxel (microtubule polymerization promoter), gemcitabine and 5-FU (DNA synthesis inhibitors), doxorubicin (Top2 inhibitor) and Cytoxan (cyclophosphamide, alkylating agent) at their respective MTD for the one time injection schedule [58] (Cao, S. unpublished data). The results revealed that FL118’s antitumor activity is superior to irinotecan and other chemotherapeutic drugs (Fig. 9).

### FL118 is able to Eliminate Patient-derived Head-&-neck Tumor Xenograft in an Animal Model

**Figure 10 pone-0045571-g010:**
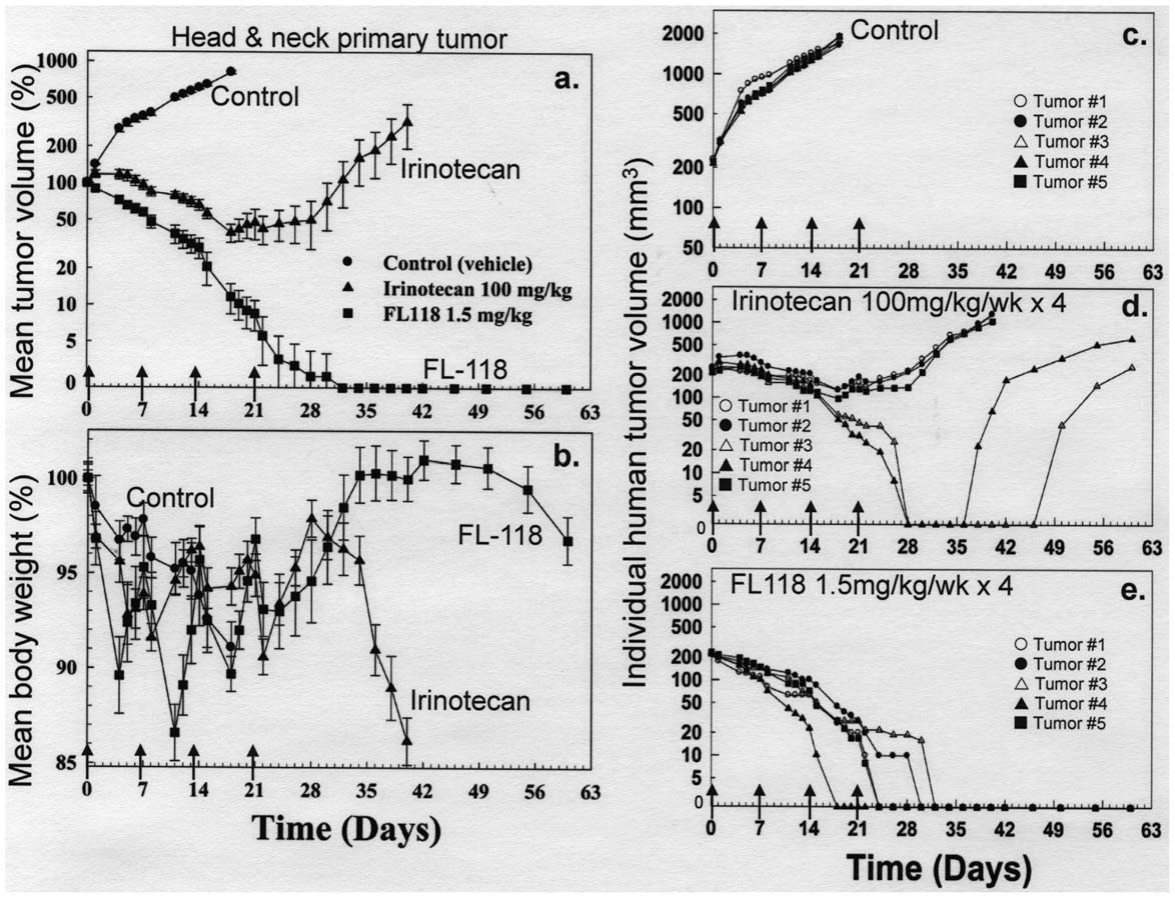
Comparison of antitumor efficacy and toxicity (body weight loss) of FL118 with irinotecan in a SCID mouse model of human primary head and neck tumor (17073)-established xenograft. The tumor model set up was the same as in Figure 9. **a**. The mean tumor growth curves ± SE derived from five individual mice in response to treatment with vehicle (control), FL118 or irinotecan. **b**. The mean mouse body weight change ± SE derived from five individual mice in response to treatment with vehicle (control), FL118 or irinotecan. **c**, **d** and **e**. Tumor growth curves from individual tumor xenografts in response to treatment with vehicle (**c**), irinotecan (**d**) or FL118 (**e**). The treatment schedule was weekly × 4 as indicated by arrows. The dose used for irinotecan and FL118 was their corresponding MTD for the weekly × 4 schedule.

Since irinotecan is structurally similar to FL118 (Fig. 1a) and irinotecan’s antitumor activity was the second best among the drugs tested (Fig. 9), we next compared the antitumor activity of FL118 with irinotecan at their MTD in a head-&-neck tumor xenograft established from a cancer patient (17073) in SCID mice using the clinically relevant schedule of irinotecan (weekly × 4). Our experiment showed that FL118 is able to eradicate human primary tumor-derived xenografts, while irinotecan could not (Fig. 10a). Specifically, two out of five mice in the irinotecan-treated group showed temporary tumor regression after treatment (Fig. 10d). In contrast, in the FL118-treated group, all five mice showed a complete response to FL118 treatment and no tumor recurred in the five mice during the two-month experimental period (Fig. 10e). The experiment further revealed that while irinotecan induced a significant and irreversible body weight loss, that induced by FL118 was temporary and reversible with rapid recovery after treatment (Fig. 10b). Additionally, FL118 was more potent than irinotecan (FL118 at 1.5 mg/kg versus irinotecan at 100 mg/kg). We further investigated the dose response of FL118 in three human cancer cell line-derived tumors with the weekly × 4 schedule. The experiments showed that FL118 induces a variable percentage of tumor regression in a dose-dependent manner, while irinotecan failed to do so even at its MTD. These results are summarized in Table 2.

**Table 2:** Antitumor activity of FL118 versus irinotecan in nude mice bearing human colon and head-&-neck tumor xenografts.

TREATMENT	ANTITUMOR ACTIVITY*			
	MTGI (%)	TDT (day)	PR (%)	CR (%)
HCT-8 (colon cancer)Control (vehicle)	–	3.3±0.4	0	0
FL-118 0.75 mg/kg	95.0±4.2	29.9±9.4	40	20
FL-118 1.00 mg/kg	90.9±4.8	41.6±12.2	60	20
FL-118 1.25 mg/kg	92.5±8.2	42.2±12.8	40	20
FL-118 1.50 mg/kg	98.3±2.1	65.2±9.8	40	50
Irinotecan 100 mg/kg	94.1±3.6	32.7±11.5	60	0
SW620 (colon cancer)Control (vehicle)	–	5.0±0.6	0	0
FL-118 0.75 mg/kg	98.8±1.6	37.5±9.6	40	60
FL-118 1.50 mg/kg	99.2±1.1	>80	40	60
**FaDu (head and neck cancer)**Control (vehicle)	–	3.6±0.4	0	0
FL-118 0.75 mg/kg	96.7±3.9	29.2±6.6	40	40
FL-118 1.50 mg/kg	100	>80	20	80

* Tumor size was measured daily for the first 4 weeks and then 3–4 time a week. The data shown here are the final result derived from the 60-day time point. MTGI: maximum tumor growth inhibition; TDT: tumor doubling time; PR: partial tumor response; CR: complete tumor response. Treatment was initiated 7 days after the tumor transplantation when the tumor volume was 200–250 mm^3^. Control mice were given vehicle solution (75% saline, 20% Tween-80 and 5% DMSO). Five mice were used in each group. All drugs were given i.p. at a weekly × 4 schedule. Data are means ± SD.

### FL118 is able to Eradicate Large Human Head-&-neck and Colon Tumor Xenografts

**Figure 11:**
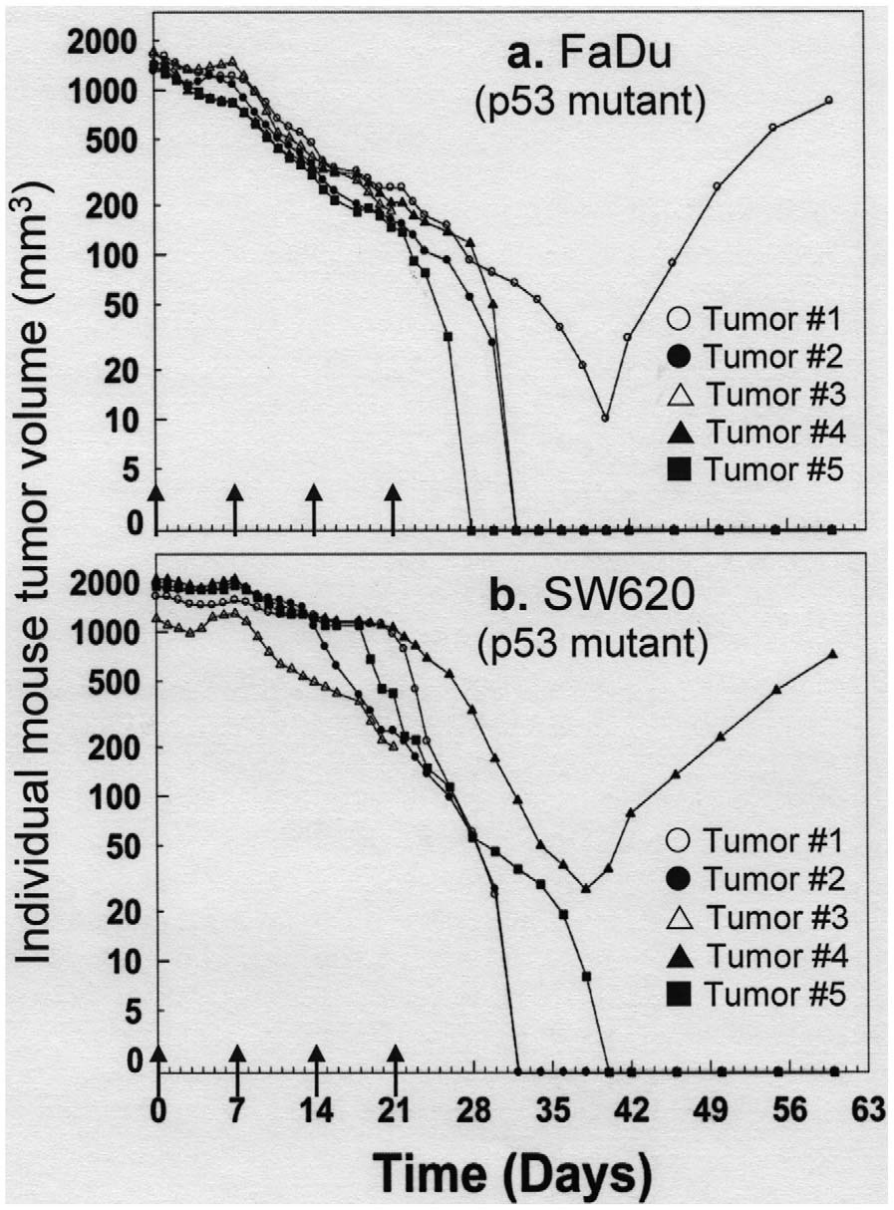
FL118 effectively eliminates large tumors. Individual athymic nude mice were xenografted with both human head and neck FaDu tumor on the left side (**a**) and human colon SW620 tumor on the right side (**b**). After the tumor grew to their maximal sizes allowed by IACUC (1500–2000 mm^3^), mice were treated with FL118 at the dose of 1.5 mg/kg weekly × 4 schedule as indicated with arrows. Each curve represents an individual mouse during and after treatment. Of note, one mouse with both FaDu (on left side, **a**) and SW620 (on right side, **b**) tumors died on Day 21 for an unknown reason. This could be the result of Tumor Lysis Syndrome (TLS), which can be caused by treatment-induced massive tumor necrosis (http://emedicine.medscape.com/article/989050-overview).

In general, advanced tumors respond poorly to chemotherapy. In contrast with this general paradigm, mice bearing the maximal human tumor volume (∼2000 mm^3^) allowed by IACUC (Institutional Animal Care and Use Committee) responded well to FL118 treatment in both human FaDu (head and neck) and SW620 (colon) cancer cell line-derived tumors. FL118 induced complete tumor regression in a high percentage (50–70%) of the mice at its MTD (1.5 mg/kg weekly × 4). A representative result from two-repeat experiments is shown (Fig. 11a, b). Thus, FL118 has the potential to effectively treat both small and large human tumors.

## Discussion

In this study, we have reported the identification and characterization of a novel small chemical molecule designated FL118 through both *in vitro* and *in vivo* studies and have ascertained the overall mechanism by which it exerts its activity. Although both FL118 and irinotecan are camptothecin analogs and camptothecin compounds are known to be DNA Top1 activity inhibitors, our data suggest that the superior activity of FL118 is not due to its greater efficacy in inhibiting DNA Top1 activity since Top1 inhibition by FL118 was less than that of SN-38, the active form of irinotecan, at the maximal achievable levels of irinotecan *in vivo* (1 µM, Fig. 1b). This suggests that other mechanisms must contribute to the greater antitumor efficacy of FL118 when compared to irinotecan (Figs. 9 and 10). In this regard, the ability of FL118 to inhibit survivin expressing in cancer cells appears to play a major role in its efficacy (Figs. 1e, f, 2, 3c, 4, 5a–c and 7).

Consistent with its initial discovery via HTS of compound libraries using the survivin gene promoter-driven luciferase reporter system as a biomarker/target [41], FL118 selectively inhibited survivin promoter activity at concentrations as low as 0.1–1 nM (Fig. 4a, b), while showing no inhibitory effects on four control promoters derived from the p21, DHFR, HTR and TK genes at 10 nM (Fig. 4c–e). Similar results were also obtained for endogenous protein expression of the relevant genes (Fig. 4f, g), indicating its selectivity. To confirm a role for survivin in FL118 activity, we knocked down survivin using lentiviral survivin shRNA in HCT-8 cells and in contrast to the results in the low/no survivin-expressing non-cancerous cells, which showed no sensitivity to FL118 treatment (Fig. 2), we found that silencing of survivin in cancer cells increased the efficacy of FL118 in inhibiting cancer cell growth (Fig. 7a), demonstrating a role for survivin in modulating FL118 sensitivity, at least in these cancer type cells. This conclusion was further confirmed by using Annexin V/PI staining and flow cytometry analysis showing that silencing of survivin significantly increased the number of FL118-induced apoptotic cells (Annexin V staining) (Fig. 7b–d). Furthermore, our data indicate that lentiviral shRNA-mediated knockdown of Mcl-1 increased (Fig. 8a), whereas overexpression of anti-apoptotic XIAP and cIAP2 decreased (Fig. 8b, c), the apoptotic response to FL118, suggesting a role for these proteins in FL118 efficacy as well. This notion was further strengthened by the experiment of Annexin V/PI staining and flow cytometry analysis after XIAP overexpression in cancer cells showing that forced expression of XIAP decreased Annexin V staining (protecting cancer cell from apoptosis, Fig. 8d–f). Additionally, the increased expression of Bax and Bim in FL118-treated cells (Fig. 5b) also would contribute to the increased apoptotic activity. Interestingly, our data demonstrated that FL118-mediated induction of Bax and Bim proteins may be through both transcriptional and posttranscriptional mechanism (Fig. 6a, b). This may or may not involve FL118-mediated inhibition of HDAC activity (Fig. 6c), which would be an interesting area for further investigation. Consistent with these findings, FL118 treatment of cancer cells inhibited BrdU label (proliferation, Fig. 3a, b) and increased the production of apoptotic markers including caspase-3 activation and PARP cleavage (Fig. 3c).

In terms of the FL118 downstream protein target relationship, since genetic silencing of survivin did not result in the modulation of other FL118 targets, including Mcl-1, XIAP and cIAP2 (Fig. 5d), it is likely that FL118-mediated inhibition of these proteins is independent of its inhibition of survivin. In this regard, we have demonstrated that FL118 not only inhibits survivin promoter activity (Fig. 4a, b) but also inhibits Mcl-1 promoter activity (Fig. 5e). Together, these findings are supported by other studies in the literature, where it has been shown that abrogation of one or more of the four genes (survivin, Mcl-1, XIAP and cIAP2) would inhibit tumor growth, sensitize drug resistant cancer cells to treatment and induce apoptosis in various *in vitro* and *in vivo* models [59]–[67]. Additionally, one interesting observation is that survivin shRNA knockdown of survivin showed a certain downregulation of XIAP and cIAP2 (Fig. 5d), although this is a very minor effect. It has been documented in the literature that different types of IAP proteins, such as survivin with XIAP [68], interact with each other, the dissociation or degradation of one protein from the complex could destabilize the other proteins in the complex. Therefore, survivin shRNA-induced silencing of survivin may induce a minor instability for XIAP and cIAP2 as an indirect secondary effect. Finally, an important consideration is that a role for each of these FL118 targeting proteins is most likely cancer cell type specific. For example, overexpression of XIAP failed to decrease caspase 3 after FL118 treatment. However, our results derived from Annexin V/PI staining revealed that overexpression of XIAP significantly decreased Annexin V-stained cells upon FL118 treatment (Fig. 8d–f). It is known that induction of apoptosis can go through multiple mechanisms and can be reflected by distinct apoptotic markers; moreover, either caspase-dependent or caspase-independent pathways (or a mix of both) may be involved. Thus, apoptosis may exhibit distinct apoptotic marker activation in a context or cell-specific manner.

As noted above, the inhibition of Mcl-1, XIAP and cIAP2 by FL118 is independent of its inhibition of survivin (Fig. 5d). A follow-up question about this result is what are the potential signaling pathways by which FL118 independently inhibits these proteins? Since we demonstrated that FL118 selectively inhibits survivin (Fig. 4a, b) and Mcl-1 (Fig. 5e) promoter activity, while showing no inhibitory effects on control gene promoters (Fig. 4c–e), we speculate that FL118 may interact with and regulate one or more transcription factors or co-factors that control the expression of the survivin, Mcl-1, XIAP and cIAP2 genes, which would be a valuable, although challenging, area for future investigation.

Although we have demonstrated a potential molecular mechanism by which FL118 inhibits cancer cell growth and induce apoptosis, the actual biochemical targets of FL118 remain to be identified. Specifically, what are the protein factors with which FL118 physically interacts after it goes into the cell? We are planning to investigate this using two complementary approaches. One is to find FL118-interacting proteins from cancer cell lysates via FL118 affinity purification, followed by mass spectrometry analysis of FL118 affinity-purified proteins displayed on one or two-dimensional gels. The other is to use tritium (^3^H)-labeled FL118 (^3^H-FL118) as a probe to screen human protein microarray to identify FL118 target proteins. Hopefully, the results from these studies will build a better foundation for further elucidation of the underlying molecular mechanism by which FL118 exerts its effects.

Based on our *in vitro* observations, we expected that FL118 should effectively inhibit tumor growth *in vivo*, since FL118 inhibited multiple cancer survival-associated genes (survivin, Mcl-1, XIAP and cIAP2). Consistent with our expectation, FL118 indeed exhibited superior antitumor activity in mouse models (Figs. 9–11). In our studies, we initially compared FL118’s antitumor efficacy with 9 clinically used chemotherapeutic agents that have different molecular mechanisms of action, in animal models of human head-&-neck (Fig. 9a) and colon (Fig. 9b) tumor xenografts. The reason for choosing colon and head-&-neck tumor was because FL118 has structural similarity to irinotecan and irinotecan has been demonstrated to be highly effective clinically in treating colon and head-&-neck cancers. Our studies showed that FL118 is superior to both irinotecan and other chemotherapeutic drugs in terms of antitumor activity (Fig. 9). In this experiment we chose the approach of one time drug injection at their one-dose MTD for initial drug efficacy evaluation and comparison with emphasis on irinotecan by using irinotecan-preferable human tumor types. We understood that while the comparison is a time and cost-effective design, this is not a perfect design from a comprehensive efficacy comparison point of view with a panel of distinct drugs, because generally speaking, different drugs have different optimal schedules and preferable human tumor types. However, we also recognized that if we compared different drugs among different tumor types with different drug injection schedules, it would raise additional issues that would need to be addressed to reach a clear conclusion. Therefore, we felt that the approach we used in the experiment shown in figure 9 matched our major focus of the FL118 and irinotecan comparison, and was time and cost-effective without significantly sacrificing the confidence of the overall data. The experiment achieved our major goal of answering whether FL118 was more effective than irinotecan.

We further investigated FL118’s antitumor efficacy in parallel with irinotecan using human primary head-&-neck tumor (17073)-established xenografts in mouse models. Using this clinically appropriate model we found that FL118 eliminates human tumor xenografts without relapse during the two-month experimental period (Fig. 10e). In contrast, irinotecan only temporarily eradicated some of the tumors with rapid relapse during the experimental period (Fig. 10d). This observation suggests that FL118 would be a better antitumor agent than irinotecan. Additionally, FL118 showed a favorable body weight loss profile in animal models (temporary and reversible), which is in contrast to the sustained body weight loss for irinotecan post treatment (Fig. 10b). We further studied the dose-dependent antitumor efficacy of FL118 in several animal models of human colon and head-&-neck cancer cell line-established tumors. The studies using these human tumor animal models revealed that FL118 is able to inhibit growth of human tumors in a dose-dependent manner over a wide range of doses (Table 2). In contrast to FL118, irinotecan failed to induce complete tumor regression even though administered at its MTD (Table 2). Our studies further revealed that FL118 effectively eradicates large human tumors in animal models (Fig. 11a, b), suggesting that FL118 may be effective in treatment of cancer patients with advanced metastatic disease. In this regard, our finding that FL118 inhibits its downstream targets (survivin, Mcl-1, XIAP, cIAP2) in a p53 status (wild type, null or mutant)-independent manner is highly significant. It is known that mutation or loss of the tumor suppressor p53 protein and simultaneous aberrant expression of one or more antiapoptotic proteins in the IAP and Bcl-2 families are important features for the advanced cancer to possess constitutive and induced resistance to treatment. For example, on one hand, inactivation of wild type p53 (mutation or loss) is a key step for later malignancy in colorectal cancer and often coincides with the transition of large adenomas into invasive carcinomas [69]. On the other hand, aberrant expression of survivin has been shown to be involved in resistance to treatment in colon cancer [14], [70]–[74]; XIAP was also shown to be a target and play a role in drug resistance in colon cancer [75]–[79]. Similar findings were reported for Mcl-1 [80] and cIAP2 [81] in colon cancer as well. Therefore, if an anticancer drug selectively inhibits the expression of survivin, XIAP, Mcl-1 and cIAP2 in a p53 pathway-independent manner, this drug would show high efficacy to selectively induce cancer cell killing regardless of p53 status. In other words, such a drug would not only be effective in killing cancer cells in early stage tumors with wild type p53 but also cancer cells from advanced stage cancer which has lost functional p53. Together, these findings warrant further development of FL118 toward clinical trial.

As mentioned in the introduction, YM155 is a promising antitumor agent, although its antitumor efficacy used as a single agent for cancer treatment is still under phase II clinical trials for a clear conclusion. In comparison with YM155, one notable difference between YM155 and FL118 is that FL118 shows superior antitumor efficacy with a weekly × 4 schedule; in contrast, YM155 needs to be subcutaneously administered as a 3–7-day continuous infusion per week for 1–2 weeks using an implanted micro-osmotic pump or given intravenously five times a week for 2 weeks in animal models [21]. Clinically, YM155 was administered by continuous intravenous infusion for 7 days followed by a 14-day rest period. Subjects were assessed for re-treatment within three days prior to the start of the next cycle [24], [25], [82]. Patients who received six treatment cycles with YM155 were considered to have a completed study [25]. Therefore, the convenient schedule for the use of FL118 provides another advantage for its further development into an effective therapeutic drug.

Our long-term goal is to develop FL118 for clinical application. To realize this goal, in addition to further elucidation of the detailed FL118 molecular mechanism by which FL118 acts, we will need to develop a more clinically relevant drug formulation and routes of its administration, study the detailed toxicology of FL118 in animal models, and determine the pre-clinical pharmacokinetics property of FL118 including a study of absorption, distribution, metabolism, and excretion. We will also need to identify the best FL118 synthetic chemistry for obtaining good manufacturing practices (GMP)-level bulk FL118. This would allow us to submit an investigational new drug (IND) to the FDA for initiating its clinical investigation.

In summary, we have identified and characterized a novel small chemical molecule showing superior antitumor efficacy in a p53-independent manner. Consistent with its antitumor activity potential, FL118 selectively inhibits multiple cancer survival-associated genes (survivin, Mcl-1, XIAP and cIAP2), while inducing proapoptotic factors Bax and Bim, and showing no inhibitory effects on control genes (p21, DHFR, HTR and TK). These findings warrant further development of FL118 toward clinical trials.
